# Supplementary figures and images for: In situ effects of simulated overfishing and eutrophication on settlement of benthic coral reef invertebrates in the Central Red Sea
Source: PeerJ. 2014 Apr 8;2:e339. doi: 10.7717/peerj.339 (PMC3994645; doi:10.7717/peerj.339)

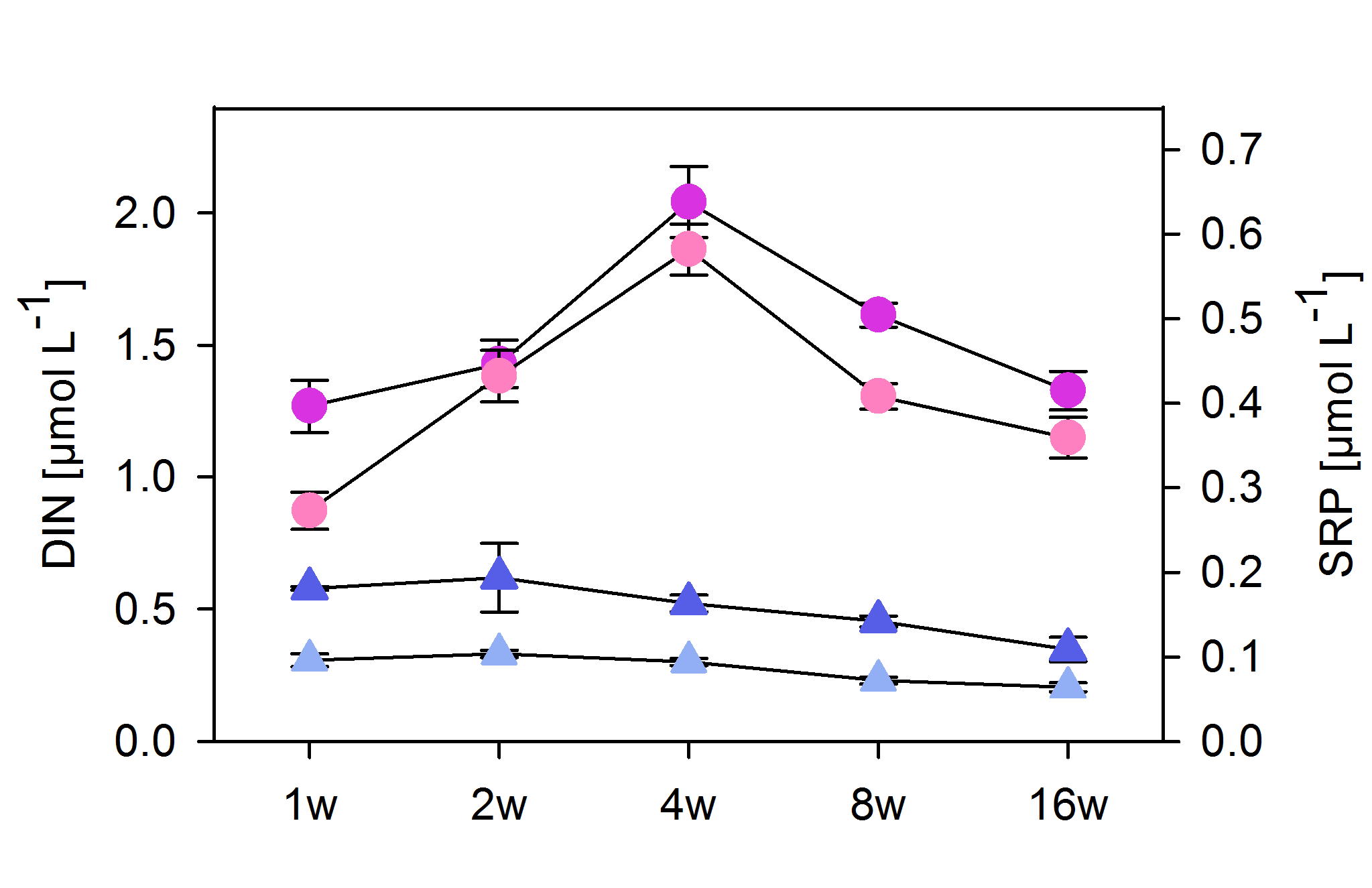

Supplement: Figure S1 — Dissolved inorganic nitrogen (DIN) and soluble reactive phosphate (SRP) concentrations (μ mol L−1; means ±SE) in the nutrient enrichment treatments (fertilizer & combined) and the non-enriched treatments (control & cage). Small letters (a—SRP; b—DIN) indicate statistical significant differences of p < 0.05 (t-test). [file peerj-02-339-s001.png]
