# Supplementary material for: In situ effects of simulated overfishing and eutrophication on settlement of benthic coral reef invertebrates in the Central Red Sea
Source: PeerJ. 2014 Apr 8;2:e339. doi: 10.7717/peerj.339 (PMC3994645; doi:10.7717/peerj.339)
Supplement: Table S1 — Abbreviations: Cage (C), Fertilizer (F), and Time (T). Significant results are indicated in bold by asterisks. P-values of 0.000 represent values < 0.001. Dashes represent factors that have been excluded by the model reduction. [file peerj-02-339-s002.docx]

|  |  |  | Scleractinia | | Bryozoa | | Bivalvia | | Polychaetes | |  |
| --- | --- | --- | --- | --- | --- | --- | --- | --- | --- | --- | --- |
|  |  | df | *F* | *p* | *F* | *p* | *F* | *p* | *F* | *p* |  |
|  | C | 1 | 6.47 | **0.013*** | 1.94 | 0.169 | 6.02 | **0.000*** | 17.21 | **0.000*** |  |
|  | F | 1 | 3.80 | 0.055 | 8.80 | **0.004*** | 8.42 | **0.000*** | 2.43 | **0.000*** |  |
|  | T | 4 | 5.84 | **0.000*** | 22.29 | **0.000*** | 33.16 | **0.000*** | 90.24 | **0.000*** |  |
|  | C x F | 1 | 14.88 | **0.000*** | - | - | 2.17 | **0.019*** | 6.05 | **0.000*** |  |
